# Utilisation of *Mangifera indica* plant extracts and parts in antimicrobial formulations and as a pharmaceutical excipient: a review

**DOI:** 10.1186/s43094-023-00479-z

**Published:** 2023-04-05

**Authors:** Mojisola Atinuke Alaiya, Michael A. Odeniyi

**Affiliations:** 1 Department of Environmental Management and Toxicology, Federal University of Agriculture, Abeokuta, Nigeria; 2 Department of Pharmaceutics and Industrial Pharmacy, Faculty of Pharmacy, University of Ibadan, Ibadan, Nigeria

**Keywords:** Medicinal plants, Antimicrobial agents, Anti-quorum sensing, Phytochemicals, Antibiotic resistance, Pectin

## Abstract

**Background:**

Antimicrobial resistance and the environmental threat posed by some synthetic antimicrobial agents necessitate more research into development of novel pharmaceutical products that are environmentally friendly. Also, the use of plant derived excipients is growing and opening up new avenue to solve current drug delivery issues in the pharmaceutical industry.

**Main body:**

This review summarizes studies related to the antimicrobial property of *Mangifera indica* extracts, possible mechanisms of antimicrobial action and antimicrobial formulations from the plant and overview of researches relating to the use of *M. indica* as a pharmaceutical excipient. Electronic searches were conducted on databases such as Pub Med, Wiley Online Library (WOL) and Google Scholar with focus on published articles relating to *M. indica*. Inclusion and exclusion criteria include publications relating to antimicrobial properties of *M. indica* extracts, its antimicrobial formulations and its use as a pharmaceutical excipient. The electronic searches yielded about 190 articles. From the studies reviewed, the mechanisms of action of phytochemicals described corroborate the antimicrobial activity exhibited by *M. indica* extracts and its selected formulations. In addition, mango pectin was observed to possess potential as a pharmaceutical excipient. Very few previous review articles based their focus on incorporating mechanism of action of phytochemicals with antimicrobial activity.

This review examined antimicrobial properties of *M. indica* extracts and formulations, major phytochemicals in the plant parts and their possible modes of action. In addition, the study assessed the use of natural polymer derived from mango plant as excipients in pharmaceutical and pharmacological preparations.

**Conclusion:**

The study concluded that effective antimicrobial activity of mango plant extracts and formulations requires synergy of actions among various phytochemical constituents of the extract or formulation. It is recommended that more researches focused on discovery of new phytochemicals in *M. indica,* their mechanisms of action and effective utilization of the plant in the pharmaceutical industry should be further explored.

## Background

Utilization of plants in ethnomedicine is in practice globally, and this has been part of history for centuries. Presently, there is great interest in the use of plants in modern pharmaceutical preparations and nutraceuticals [1]. The issue of antimicrobial-resistance, brought about by resistance of certain bacteria to known antibiotics, is a threat to global public health and constitutes an obstacle to effective prevention and treatment of diseases [2, 3]. According to Okareh et al. [4], antimicrobial resistance and the environmental threat posed by some synthetic antibacterial agents necessitate more research into development of novel antimicrobial formulations that are environmentally friendly. The bioaccumulation and persistence of the active agents in some conventional antimicrobial preparations in the environment have been reported to impact adversely on the ecosystem and human health [5, 6]. Triclosan has been linked to environmental pollution and endocrine disruption [7]. Chlorhexidine is reported to possess genotoxic and cytotoxic effects on human lymphocytes [8]. Hexachlorophene, the active pharmaceutical ingredient in many antiseptic personal care products, has been found to be resistant to biotransformation. This resistant has led to its accumulation in the environment and inadvertently the food chain [9].

A number of review articles focused on pharmacological properties of mango plant parts, and the phytochemicals they contain have been published. Jahurul et al. [10] reviewed the use of mango seed and peel in the food industry. The study also examined the activity of the bioactive compounds in the plant parts. The study reported that mango seed and peel contain mainly phenolic compounds such as mangiferin, flavonoids and tannins. According to Jahurul and colleagues, the antimicrobial characteristics of the plant parts of interest are due to the presence of these phenolic compounds. It was concluded in this study that more researches on the specific applications of these plant parts will encourage industrial exploitation.

Parvez et al. [11] reviewed the pharmacological activity of *M. indica* leaves, flowers, fruits, seed kernel, bark and roots. The study concluded that the plant possesses potential as an antimicrobial agent and this was ascribed to its leaves and stem bark. In addition, the study attributed anticancer property to mango fruit juice, anti-inflammatory property to its stem bark and anti-diabetic property to the leaves and stem bark. Ediriweera et al. [12] reviewed the pharmacological activities of *M. indica* plant parts (leaves, fruit, flower, stem bark and roots) and their applications in ethno-medicine. Mangiferin, tannins and other major phenolic compounds were found to be the predominant secondary metabolites in the various plant parts. The study attributed *M. indica*’s antimicrobial activity to the presence of these secondary metabolites (tannins, mangiferin and quercetin). Antioxidant activity was reported to be the main pharmacological property of the plant part extracts. Ediriweera and colleagues reported that the antimicrobial properties exhibited include anti-plasmodial, antibacterial, antifungal, antiviral and antimalarial activities. Antiviral activity was not reported for quercetin. The review also reported that mango leaves and kernel were the most investigated parts for antimicrobial effect among the studies considered.

Lebaka et al. [13] reviewed the pharmacological property of mango pulp, peel and kernel. The study reported the presence of bioactive compounds with antimicrobial, anticancer and anti-inflammatory effects in the plant part extracts. Lebaka and colleagues concluded that these phytochemicals have nutritional and therapeutic benefits which can be useful in the food industry. However, the possible modes of action of the bioactive compounds were not reported.

These previously published reviews did not report explicit possible mechanisms of action of the phytochemicals attributed with the pharmacological effects of the plant parts. In addition, very few reviews focused on antimicrobial formulations from mango plant part extracts and the use of the plant as a pharmaceutical excipient.

In this review, possible mechanisms of action of phytochemicals reported to possess antimicrobial properties in mango plant part extracts and formulations were considered. Specific mechanisms of action of some major phytochemicals were elucidated to understand their importance in ethno-medicine and antimicrobial preparations. Antimicrobial formulations were categorized based on their utilization. In addition, utilization of *M. indica* as a pharmaceutical excipient and in therapeutic preparations was also considered.

### Mangifera indica

Mango is an evergreen broad canopy tree. The plant is a member of the family Anacardiaceae and genus Mangifera consisting of about sixty-nine different species. It is the most common specie in the genus, and it is considered to have originated from Asia but cultivated in different countries of the world [12, 14]. *M. indica* is reported to contain most of the major known phytochemicals [4, 15, 16]. Diso et al. [15] reported the presence of alkaloids, phenols, flavonoids, saponins, tannins in the leaf and stem bark of the plant. Steroids were found to be present only in the stem bark, while anthraquinones were found only in the leaves. A study conducted by Sarker and colleagues [17] on mango fruit pulp reported the presence of alkaloids, flavonoids, saponins and tannins. Okareh et al. [4] reported abundant presence of tannins and phenols in both mango kernel and leaf extracts. Saponins, flavonoids, alkaloids and terpenoids were also reportedly present. Ogidi et al. [18] reported the presence of saponins, flavonoids, tannins, glycosides, alkaloids, terpenoids and steroids in the stem bark of *M. indica*.

Phytochemicals are reported to be the scientific basis for utilization of plant parts for the treatment of diseases in ethnomedicine [12]. *M. indica* is an ethno-medically diverse plant with various uses in the treatment of different diseases traditionally across the globe [12]. Mango plant parts (leaves, fruit, seeds, kernel and bark) are reportedly used in ethno-medicine globally.

This review summarizes studies related to the antimicrobial property of *M. indica* and its formulated antimicrobial preparations. Additionally, this paper presents an overview of the use of *M. indica* as a pharmaceutical excipient.

### Pathogenic bacteria and antimicrobial resistance

According to the World Health Organization [3], pathogenic bacteria are the major cause of diseases and death in developing countries. The WHO stated that twelve pathogenic bacteria of public health importance are increasingly posing great threat to human health because of their resistance to known antibiotics. These bacteria include *Staphylococcus aureus*, *Escherichia coli* sp., *Salmonella* sp. and *Pseudomonas aeruginosa* [3]. Antibiotic resistance is a public health problem that has created a new burden on modern healthcare [19, 20]. The emergence of antimicrobial-resistant bacteria threatens the effective prevention and treatment of diseases [2]. Antimicrobial resistance has been declared a global public health issue of international concern (PHIC) by the WHO. The Food and Agricultural Organization (FAO) [21] stated that antimicrobial resistance occurs when microorganisms (pathogenic bacteria, fungi, protozoans and viruses) become increasingly less susceptible to the inhibitory or microbicidal activities of antimicrobial drugs or antibiotics.

## Methods

Electronic searches were conducted on databases such as Pub Med, Wiley Online Library (WOL) and Google Scholar. About 4176 hits were found on PubMed and 10,000 on WOL. About 190 publications were retrieved from the search. Search terms such as “Mangifera indica”, “medicinal chemistry”, “polyphenols”, “phytochemical”, “antimicrobial resistance”, “medicinal plants”, “mango antimicrobial formulations”, “mango excipients”, “antimicrobial agents”, “excipients”, “natural polymers” and other related words or phrases were used. In total 74 peer-reviewed journal articles, 6 web resources, 2 conference papers from proceedings and 3 book chapters were utilized in the study. Peer-reviewed journals and other articles aimed at ethno-pharmacological studies of *M. indica*, its antimicrobial assessment, therapeutic and pharmaceutical uses were considered to be eligible materials for writing this review.

### Inclusion and exclusion criteria

- Publications relating to antimicrobial property of *M. indica*
- Published researches involving antimicrobial formulations from mango plant parts
- Published articles relating to natural polymers obtained from *M. indica* and their use as pharmaceutical excipients.
- Published works in English including theses, articles and proceedings that dealt with antimicrobial activity of mango extracts, formulations from the extracts and natural excipients.

### Antimicrobial activity of *M. indica* plant part extracts

Various studies have been conducted on extracts obtained from leaves, fruit peel, root, stem bark and seed kernel of *M. indica* to ascertain their antimicrobial activities. Quantity of extracts and quantity of phytochemicals in a particular extract are dependent on a number of factors which include type of solvent used, extraction method, seasons and environmental conditions [22]. These factors are important because some phytochemicals can be denatured by heat and certain solvents.

Ogidi et al. [18] investigated the antimicrobial properties of aqueous and methanol extracts of stem-bark of *M. indica* against selected microbes namely *Shigella sp, Staphylococcus sp, Escherichia coli, Vibrio sp, Penicillium* sp.*,* yeast and mould isolates using agar well diffusion method. The study reported that *M. indica* stem bark exhibited antimicrobial activities against all test organisms. Zone of inhibition (ZOI) of the methanol extract was found to be highest for *Staphylococcus* sp. (15.4 ± 0.36 mm) among the bacterial group and for *Penicillium* sp. (9.3 ± 0.2 mm) among the fungi group. ZOI of aqueous extract was found to be highest for *E. coli* (10.6 ± 0.2 mm) among the bacterial group and *Penicillium sp* (10.3 ± 0.3 mm) among the fungi group.

Bshabshe et al. [23] investigated the antibacterial activity of ethanol extract of mango kernel on clinical isolates of *S. aureus* and methicillin-resistant *S. aureus* (MRSA) using in vitro disc diffusion method. The antibacterial activity of the extract was compared to that exhibited by standard antibiotics (vancomycin, ampicillin and penicillin). The study reported that mango kernel extract demonstrated antibacterial activity against both categories of bacteria. MRSA strains were found to be more sensitive to mango kernel extract when compared to the reference drug vancomycin. Further, the study reported that MRSA showed 100% sensitivity to vancomycin, 100% resistance to ampicillin and 98% resistance to penicillin.

Osei-Djarbeng and colleagues [24] conducted a study to investigate and compare the antimicrobial property of ethanol extracts of mango bark, leaves and seed against selected microbes using broth dilution and agar well diffusion analytical methods. The study reported that the seed extract exhibited the highest antimicrobial activity among the extracts. Minimum inhibitory concentration (MIC) of seed extract against *S. aureus* was reported to be 125 µg/mL.

Diso et al. [15] assessed the antibacterial activity of mango stem and leaf extracts on methicillin-resistant *S. aureus*. The study reported that the stem extract exerted higher inhibitory activity against the bacteria isolate compared to the leaf extract. Mutua and colleagues [25] explored the antimicrobial activity of mango seed powder extracts of four mango cultivars against *S. aureus, Candida albicans, Bacillus subtilis, P. aeruginosa* and *E. coli*. The extracts demonstrated highest activity against *C. albicans* compared to other test organisms. The researchers concluded that the extracts exhibited greater antifungal activity in comparison with antibacterial activity.

Manzur et al. [26] assessed the antimicrobial activity of mango leaf extract on eight *S. aureus* strains isolated from a cow with mastitis disease and *S. aureus* strain ATCC 25923. Aqueous and ethanol extracts of the leaves were prepared via maceration method of extraction. Phytochemical analyses showed the presence of tannin and other phenolic compounds. The study reported that ethanol extract exhibited higher antimicrobial activity against the test organisms compared to the aqueous extract. *S. aureus* strain ATCC 25923 showed highest susceptibility to the extract (MIC between 1.8–7.5 mg/ml and MBC between 15.1–45.3 mg/ml). All the *Staphylococcus *spp. strains formed biofilms on stainless steel cup and teat rubber surfaces with cell count values greater than 7 log CFU/cm^3^. The surfaces were then subjected to ethanol extract at 45.3 mg/ml. The study reported 100% reduction in bacterial biofilm formation after contact with the ethanol extract. *S. aureus* strain SA178 was found to demonstrate the highest susceptibility to the extract.

Adepiti et al. [27] investigated the in vivo anti-malarial activity of a decoction made with powdered leaves of mango and other plants on *Plasmodium berghei* infested mice model. The study reported 55% *P. berghei* reduction by the fifth day of treatment.

Disegha and Akanni [28] investigated the antifungal activity of *M. indica* leaf extract against *Aspergillus fumigatus, A. flavus, A. niger* and *C. albicans*. The antifungal activities of cold aqueous (maceration), hot aqueous (decoction) and ethanol extract were compared at 40% concentration. The hot aqueous extract exhibited highest antifungal activity against all test organisms. *C. albicans* was found to be most susceptible to the extract with ZOI measurement of 18.0 ± 2.0 mm. Mango leaf ethanol extract demonstrated the least inhibitory activity against all test fungi. The study concluded that *M. indica* extracts can be used as antifungal agent (Table 1).

**Table 1:** Summary of mango plant part extracts and their antimicrobial effects on selected microbes

Plant part	Extraction method	Solvent of extraction	Most susceptible organisms of interest	Highest antimicrobial activity ZOI(mm) or MIC(mg/mL)	
Stem bark	Maceration	Methanol Aqueous	*Staphylococcus* sp. Penicillium sp. *E. coli* *Penicillium* sp.	15.4 ± 0.36 (methanol extract) 9.03 ± 0.2 (methanol extract) 10.6 ± 0.2 (aqueous extract) 10.3 ± 0.3 (aqueous extract)	[18]
Kernel	Soxhlet	Ethanol	MRSA *S. aureus*	20.77 ± 0.61 @50 mg/ml	[23]
Seed Leaf Stembark	Maceration	Ethanol	*S. aureus* *S. faecalis* *E. coli* *K. pneumonia* *C. albicans*	19 ± 1.0 17 ± 0.3 17 ± 0 18 ± 1.0 14 ± 1.0 (Seed extract)	[24]
Leaf Kernel	Maceration	Methanol	*S. aureus* *Salmonella* sp. *E. coli*	20.70 ± 1.05 15.20 ± 01.04 16.20 ± 0.96 (Kernel extract)	[4]
Leaves	Maceration	Methanol Water	*S. aureus* isolates *S. aureus* strains	MIC 1.8–7.5 & MBC 15.1–45.3	[26]
Leaf Stem	Maceration	Water chloroform	MRSA	17.0 aq.leaf extract @ 120 mg/ml	[15]
Leaves	Decoction	Water	*C. albicans* *Aspergillus flavus* *A. niger* *A. fumigatus*	18.0 ± 2.0 12.1 ± 2.0 11.0 ± 1.0 8.33 ± 0.58 @40 mg/ml	[28]
Leaves	Decoction	Water	*Plasmodium berghei*	55% *P.berghei* reduction	[27]

### *Mangifera indica*: possible mechanisms of antimicrobial activity

A number of studies have ascribed antimicrobial properties of *M. indica* to the presence of phytochemicals such as tannins, alkaloids, flavonoids, mangiferin and phenolics [16]. The degree of antimicrobial activity is reported to be dependent on extraction solvent and method of extraction of plant parts containing these secondary metabolites [22, 26]. The antimicrobial effects exhibited include antibacterial, antiviral, antifungal and anti-plasmodial activities.

### Antibacterial effect of mango

It is noteworthy that antibacterial activity is not exclusive to only a particular phytochemical. More than one secondary metabolite can exhibit antibacterial effect. Some studies have attributed antibacterial activity of plant extracts largely to the presence of tannins [29, 30]. However, it has been found that effective antimicrobial activity of plant extracts involves synergy among different phytochemicals.

### Antibacterial activity of tannins

Collectively, tannins are plant polyphenols with phenolic hydroxyl groups on a phenyl ring [29]. They are classified into two main groups namely hydrolysable tannins (e.g. gallotannin) and condensed tannins (e.g. proanthocyanidin) [31]. Engels et al. [30] isolated gallotannin from mango kernel extract and investigated the antibacterial mechanism of action on *Bacillus subtilis*. The study reported that gallotannin forms an unstable complex with iron which favours oxidation of Fe^2+^ to Fe^3+^.

**Fig. 1:**
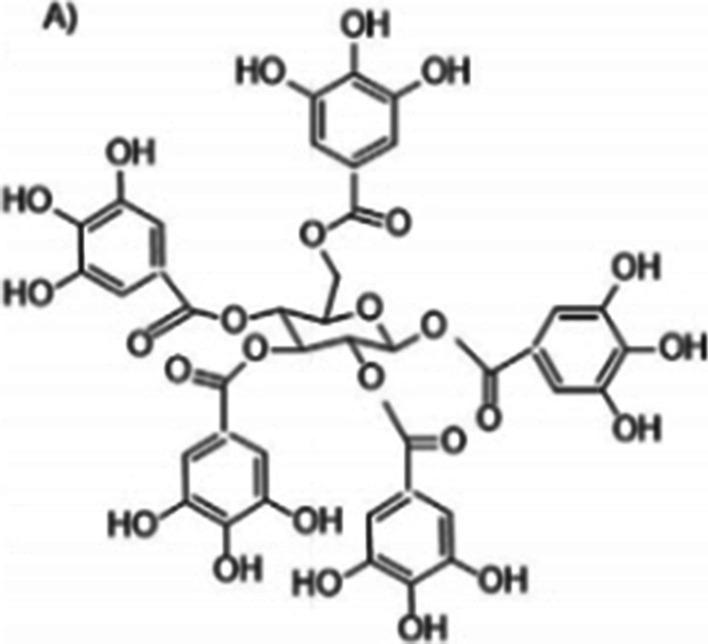
Structure of a simple gallotannin. Source: [30]

Tannins also have the ability to precipitate protein from bacteria, and this property is due to their possession of a number of hydroxyl groups which aid their function as protein binders. Thus, the antibacterial activity of gallotannin is attributable to its strong affinity for iron and inactivation of membrane-bound proteins [31]. Engels and colleagues found that Gram positive bacteria are more susceptible to gallotannin compared to Gram negative bacteria. According to the researchers, the charged outer membrane of Gram-negative bacteria may limit binding of the negatively charged gallotannin through electrostatic repulsion (Fig. 1).

### Antibacterial activity of mangiferin

Mangiferin is reported to be the most abundant polyphenol in *M. indica* extracts. It is a glucoxanthone reported to exhibit antibacterial and other antimicrobial effects [32].

**Fig. 2:**
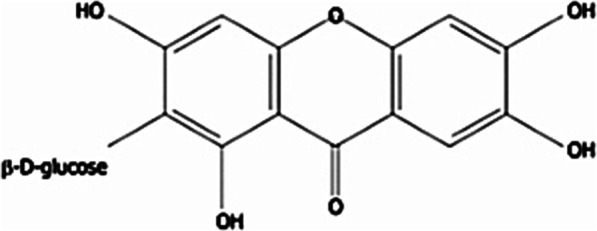
Chemical structure of mangiferin. Source: [35]

**Fig. 3:**
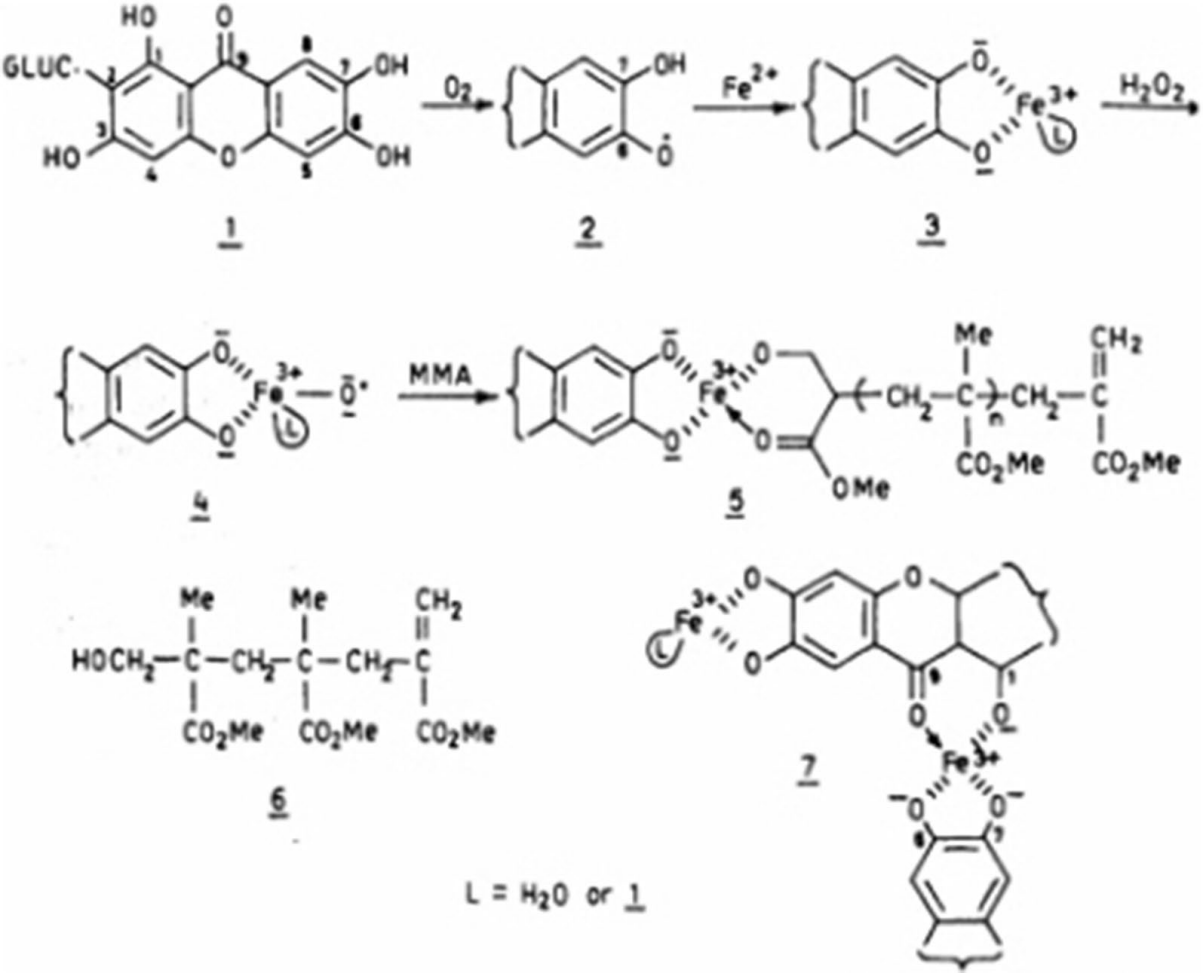
Chemical representation of possible mechanism of action of mangiferin. Source: [36]

Although mangiferin is known much more for its anti-diabetic/anticancer/antioxidant properties, it possesses a significant antibacterial activity [33]. Mangiferin exhibits antibacterial activity against both Gram positive and Gram-negative bacteria. Singh et al. [34] reported antibacterial activity of mangiferin isolated from ethanol extract of mango stem bark against Gram positive *B. pumilus, B. cereus* and Gram-negative *Salmonella virchow* (Figs. 2, 3).

### Antibacterial activity of alkaloids

**Fig. 4:**
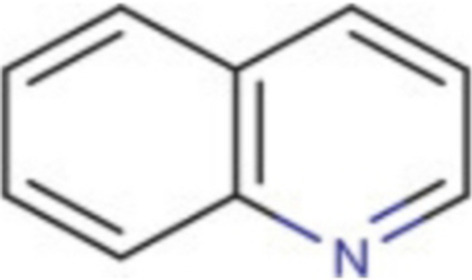
Basic quinoline structure (an alkaloid). Source: [37]

Alkaloids are a group of phytochemicals characterized by great structural diversity with the presence of a basic nitrogen atom as the only unifying feature [16, 37, 39]. Alkaloids readily form hydrogen bonds with proteins, enzymes and receptors due to their possession of a proton-accepting nitrogen atom and one or more proton-donating amine hydrogen atoms. Alkaloids exhibit their antibacterial property through a number of mechanisms as follows:

(i) Inhibition of nucleic acid synthesis
(ii) Inhibition of the enzyme *dihydrofolate reductase*
(iii) Inhibition of bacterial efflux pump
(iv) Inhibition of both biofilm formation and production of virulence factors through anti-quorum sensing activities [16] (Fig. 4).

### Antiviral activity of phytochemicals in mango: flavonoids

Flavonoids are polyphenols consisting of phenolic and pyran rings with hydroxyl, methoxy and glycosidic functional groups. Flavonoids found in mango include quercetin, catechin and epicatechin. They are known for their antioxidant capacity and scavenging activity against reactive oxygen species and free radicals. Their pharmacological activity is enhanced by their ability to penetrate microbial cell membrane. Flavonoids have been reported to show antiviral activity against *Herpes simplex* virus, respiratory syncytial virus, *Parainfluenza* virus and *Adenovirus* [40, 41].

**Fig. 5:**
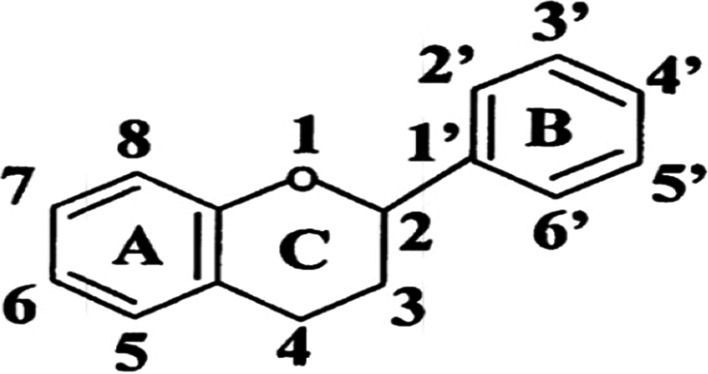
Basic nuclear structure of flavonoids. Source: [42]. Pharmacological activities of flavonoids and their metabolites in vitro depend upon the arrangement of functional groups about the nuclear structure

According to Nijveldt [40], flavonoids exhibit antiviral property by inhibiting different stages in the replication cycle of viruses. This is achieved via two mechanisms: (i) inhibition of intracellular replication of viruses and (ii) inhibition of infectious properties of viruses (Fig. 5).

### Anti-HIV and other antiviral activity of mangiferin

Wang and colleagues [43] investigated the anti-HIV-1 activity of mangiferin. The study reported that mangiferin exerts its mechanism of action via inhibition of the HIV-1 protease. Mangiferin was also found to antagonize the in vitro cytopathic effect of HIV on MT-2 cells and prevented cell death. According to Singh et al. [34], the anti-HIV activity is possibly associated with mangiferin’s characteristic as an interferon inducer.

### Antifungal effect of *M. indica* phytochemicals: mangiferin

Mangiferin has been found to exert antifungal activity against *C. albicans* via inhibition of biofilm formation [44]. A study conducted by Singh et al. [34] reported that mangiferin exhibited antifungal activity against *T. aurantiacus* and *A. flavus*. Gallotannin is unable to inhibit the fungi *Aspergillu*s sp. and *Penicillium* sp. due to the synthesis of the enzyme *Tanase* which has the ability to degrade tannins [30].

## Mechanism of action of mango phytochemicals

Based on the studies considered, the possible mechanisms of action of *M. indica* phytochemicals are summarized below.

### Complexation of iron (Fe) needed by microbes

All the major phytochemicals, e.g. tannins, flavonoids (quercetin), mangiferin are reported to possess iron chelating property. According to Engels et al. [30], the phytochemical gallotannin possesses strong affinity for iron and readily binds to the metal on microbial cell surface forming a complex. Iron is needed for important metabolic activities by microbes; hence, they secrete biomolecules known as siderophores which aid in sequestration of iron for their use.

Siderophores have high affinity for iron and bind the metal to form ferrisiderophores. Gallotannin is structurally similar to siderophore; hence, it binds the membrane receptor of the microbe in its place. This deprivation of the much needed iron results in inhibition of microbial growth and ultimately cell death. Complexation of iron also results in precipitation of microbial proteins through interaction with cell membrane.

### Inhibition of quorum sensing (QS) system in microbes

QS is a gene regulatory system that coordinates genetic expression. QS mechanism involves synthesis, release and uptake of auto-inducers among other processes in microbes [45]. Auto-inducers are extracellular signalling molecules synthesized by microbes for regulation of genes encoding bacterial expressions such as virulence, motility and proteolytic enzymes secretion. Hussain et al. [45] in their study tested different extracts of mango leaf (petroleum ether, benzene, ethyl acetate, acetone and methanol) against *C. violaceum* strain CV12472 for QS modulatory activity. Pyrogallol was reported as the main phytochemical in *M. indica* leaf extracts. The study recorded that QS inhibitory ability of the extract was dose dependent. Hussain and colleagues concluded that mango leaf extract possesses the ability to disable the QS system of *C. violaceum* strain CV12472. According to Asfour [46], QS system inhibition can be effected through:

(i) Inhibition of synthesis of auto-inducers.
(ii) Antagonism of auto-inducer receptors
(iii) Degradation of auto-inducers among others.

Other effects resulting from inhibition of quorum sensing system include:

- Inhibition of biofilm formation
- Neutralization of bacterial toxins

### Damage to microbial cell membrane

Raybaudi-Masillis et al. [47] reported that phenols cause damage to microbial cell membrane by interacting with microbial enzymes. The process was reported to involve adsorption of phytochemicals by the cell membrane. This adsorption of phenolic compounds causes alteration of pH and electrical potential of the microbial cell membrane. Damage to microbial cell membrane leads to (1) leakage of cytoplasmic material and (2) cell death.

### Disruption of microbial enzyme synthesis

Phytochemicals also exhibit their antimicrobial ability via inhibitory activities on microbial enzyme synthesis. Bodiba et al. [48] conducted a research on the mechanism of action of mango leaf ethanol extract on *S. mutans.* They reported that the synthesis of the GTF enzyme encoded for by the gtfß gene was disrupted in the presence of the extract. *S. mutans* gtfß mRNA degree of expression in the presence of the mango leaf extract was compared to that without the extract using reverse transcription polymerase chain reactions. It was reportedly found to be lower in the presence of the extract**.**

### Interference with the replication cycle of viruses

Another mode of antimicrobial action reportedly demonstrated by phytochemicals is inhibition of viral replication cycle. According to Nijveldt [40], flavonoids inhibit various stages of the replication cycle of target virus via (1) inhibition of the intracellular replication of the virus and (2) inhibition of the infectious properties of the virus. However, the study reported that most studies focused on inhibitory activity of reverse transcriptase or RNA-directed DNA polymerase. In addition, it was reported that the investigations were carried out in vitro and thus, the scope of findings was limited.

### Modification of microbial membrane properties

Gallotannin is reputed to possess the ability to form complexes with proteins. The mechanism of this action has been found to be in two stages. First, it binds to microbial protein. Secondly, it causes aggregation of the microbial proteins resulting in precipitation [31]. These effects cause modification of microbial membrane properties and change in microbial cell membrane fluidity.

### Antimicrobial formulations from *M. indica* extracts

Phytochemicals are reputed to confer pharmacological attributes on plants. Several researches have been conducted to validate ethno-pharmacological claims of the medicinal efficacy of mango plant part extracts. *M. indica* plant part extracts are reported to possess antimicrobial properties. Based on this claim, studies have been conducted to formulate antimicrobial therapeutic preparations from the plant extracts. These formulations include skincare products, nutraceuticals, hygiene products, pharmaceuticals among others.

### Mango antiseptics ointments, cream and lotions

Okareh and colleagues [4] formulated antimicrobial ointments from mango kernel, leaf and guava leaf methanol extracts and investigated their antibacterial activities against *S. aureus, E. coli* and *Salmonella *sp. The ointments were formulated according to the British Pharmacopoeia procedure. Each plant extract (50 mg) was incorporated into ointment bases, respectively, to produce 5% (w/w) antiseptic ointment preparations. Microbiological assay was conducted using agar dilution and diffusion techniques. The analyses were conducted in duplicates. Gentamicin (10 mg/L) was used as the reference drug while ointment base was used as the negative control. The differences in mean zone of inhibitions (ZOI) among the extracts and among ointment formulations against all organisms of interest in the study were reported to be significant. Mango kernel extract and formulation were found to exhibit highest inhibitory activity. In addition, *S. aureus* was reported to exhibit the highest susceptibility to the plant extracts and ointment preparations. MIC for *M. indica* kernel ointment was found to be 25 mg/mL for *S. aureus, E. coli* and *Salmonella sp*. In addition, the researchers found that mango kernel extract and ointment exhibited highest significant antibacterial activity (20.70 ± 1.05 and 18.00 ± 0.89, respectively) against *S. aureus*. It was concluded that *M. indica* kernel ointment exhibited highest antibacterial efficacy among the ointment formulations. In addition, the kernel ointment was reported to possess antibacterial activity against *S. aureus* comparable to gentamicin the reference antibiotic.

Ningsih et al. [49] formulated antifungal ointment from mango leaf methanol extract and investigated its antifungal activity against *C. albicans* using agar diffusion method. Ketoconazole was used in the study as reference drug (positive control) while ointment preparation without extract was the negative control. The result of ZOI obtained at 30, 65 and 125 ppm of ointment formulation was reported to be 3.07 mm, 5.96 mm and 9.51 mm, respectively. The ZOI for positive control was reported to be 14.13 mm. The pH value of 6.56- 6.99 was reported. Another study by Ningsih et al. [50] was conducted to formulate an antibacterial ointment from mango leaf methanol extract. The inhibitory activity of the ointment was assessed against *Propionibacterium acnes* using the agar diffusion method which was indicated by measurement of the ZOI for 15 days. The pH of the ointment was reported to be 4.92–5.87. The researchers reported that ZOI for day zero at 15 ppm was 23.60 mm and 21.88 mm for day 15. The research concluded that the growth of *P. acnes* was inhibited by mango leaf extract and formulated ointment.

A study by Rao et al. [51] was conducted to formulate a topical gel from mango leaf methanol extract. 100 mg (F1), 200 mg (F2) and 300 mg (F3) of extract were used in the preparation of the ethosomal topical gel formulations 1, 2 and 3, respectively. Antibacterial activity of the gel formulations was assessed against *P. aeruginosa* and *B. subtilis* using agar well diffusion technique. Ciprofloxacin and norfloxacin were used as reference drugs. The formulations were reported to have pH range between of 5.4–6.2. The drug content of gels ranged between 74.67% and 82.31%. The study reported that formulation 2 exhibited highest antibacterial activity among the formulations with ZOI of 9 mm for *B. subtilis* and 13 mm for *P. aeruginosa*.

### Body scrub, soap and disinfectants

Bahari and colleagues [52] formulated two body scrubs using mango seed flour. Methanol extract was obtained from Perlis Sunshine mango seed flour and used to formulate rough salt and oil-in-water (o/w) scrubs, respectively. One gram (1 g) of extract was incorporated into 100 g of base. Body scrub without the seed flour extract was used as control. HPLC was used to measure ascorbic acid content in the body scrubs. The study found that the oil-in-water mango seed flour scrub possesses highest total phenolic content (6.65 ± 0.16 mg/g) and better antioxidant potential (32.29 ± 2.60% of DPPH activity inhibition) among the body scrubs (rough salt formulation, oil-in-water and control). The rough salt scrub was reported to have highest ascorbic acid content.

Rodriguez-Fernandez and colleagues [53] formulated liquid soap from mango kernel and seed oil extract. The soap was prepared from 3 g of the seed oil and 0.5 M sodium hydroxide solution via saponification. The antimicrobial activity of the formulation was assessed against *Candida auris, E. coli, S. aureus* and *P. aeruginosa* via the disc diffusion technique. Commercial cleaning/disinfecting agent containing isopropyl alcohol was used as control. The cleaning efficiency of the extract formulation was determined by image analysis with different surfaces such as glass, metal and wood. The research reported that the extract formulation did not show significant antimicrobial activity to the test organisms. However, the formulation was reported to demonstrate high comparable cleaning efficiency.

### Oral hygiene formulations

Dandekar and Winnier [54] conducted a research to assess the antibacterial activity of mouthwashes prepared from mango and neem twigs extracts against *Streptococcus mutans*. The formulations were prepared from 25% extract concentration, respectively. Chlorhexidine (0.2%) was used as the positive control. Antibacterial activity was determined via the agar diffusion technique. Zone of inhibitions of extracts were reported to be 19 mm for mango extract and 18.5 mm for neem extract at 25% concentration. The study concluded that mango and neem formulations exhibited satisfactory antibacterial activity against *S. mutans*. In addition, anti-gingival and anti-plaque activities were reported to be comparable with that of chlorhexidine the reference drug. This report is in contrast to findings reported by Bhat et al. [55].

Bhat and colleagues [55] formulated mouthwash from mango leaf extract and evaluated antimicrobial property on growth of *Streptococcus* spp*.* in salivary secretion of volunteers. Anti-gingival inflammation and anti-plaque accumulation activities were also evaluated. The susceptibility of test organisms (*S. mutans, Streptococcus mitis*, and *Streptococcus salivarius*) in saliva to the mouthwash was compared to that of chlorhexidine, the reference drug. The study reported that both the *M. indica* leaf mouthwash (2%) and chlorhexidine mouthwash (0.12%) inhibited growth in microbial population in the test samples. Reduction in plaque build-up and improved gingival health were also recorded. Chlorhexidine mouthwash was reported to exhibit significantly higher efficacy in comparison with mango leaf mouthwash. According to the researchers, chlorhexidine causes dental staining and possesses cytotoxic effects. *M. indica* mouthwash reportedly exhibited no side effects.

Anand et al. [56] conducted a research to assess the possible utilization of *M. indica* in oral healthcare. Mango plant and guava plant leaves were extracted, respectively, via maceration method. Ethanol was used as the extraction solvent. 2 mg/mL of each plant extract was used to prepare the oral hygiene formulation. In vitro antimicrobial activity of extracts was investigated against *Enterococcus faecalis*, *E. coli, C. albicans, S. mutans* and *S. aureus*. Residin (0.2% chlorhexidine) and povidone-iodine-based mouth rinse were used as positive control and reference drugs. Ethanol was used as negative control. The susceptibility of the test organisms to plant extracts was determined via agar well diffusion technique. The minimum inhibitory and bactericidal/fungicidal concentrations (MBC/MFC) were determined by micro-dilution technique with slight modification. The study reported that *E. coli* exhibited the highest susceptibility to *M. indica* leaf extract (ZOI = 20.33 ± 0.57) among the test organisms. Anand and colleagues also reported that mango leaf extract exhibited antibacterial activity higher than chlorhexidine (ZOI = 15.67 ± 0.57).

Sekar and Abdullah [57] formulated herbal toothpaste from mango, lemon and pomegranate peelings’ methanol extracts. Five grams (5 g) of each extract was incorporated into base for the formulation. Antimicrobial activity of the herbal toothpaste (with 100 mg, 250 mg and 500 mg of toothpaste base, respectively) was investigated against *P. aeruginosa, E. coli*, *B. cereus* and *S. aureus* via disc diffusion technique. Ciprofloxacin was used as the reference drug. The study reported that *S. aureus* showed the highest sensitivity to the formulated toothpaste among the test organisms. The positive control ciprofloxacin exhibited significantly higher antimicrobial activity against *P. aeruginosa, E. coli*, *B. cereus* and *S. aureus* compared to the extract formulation. The study concluded that *M. indica* peeling extract possesses promising antimicrobial effect against the organisms of interest in the study.

### Wound healing formulations

Espinosa-Espinosa et al. [58] researched the effect of methanol extract of *M. indica* peel on incision wounds in a murine model using 5% dexpanthenol as positive control. According to the researchers, the incision model was used to assess the healing efficiency of the plant extract. The healing efficiency assessment includes observation of wound contraction, tensile strength, scar formation and histological analysis. Antibacterial activity of the extract was investigated against *S. epidermis, P. aeruginosa*, *E. coli* and *S. aureus*. Acute dermal toxicity and anti-inflammatory property of the extract were also investigated.

The study reported that there was no significant difference in wound contraction closure of mango extract-treated incision wound and dexpanthenol-treated incision. The same observation was reported for the analysis of tensile strength of the incisions.

In addition, it was reported that histological assessment of the mango extract and dexpanthenol-treated incisions showed similarity in architecture. Further, the study reported that *M. indica* extract exhibited antibacterial activity against all test organisms. The highest antibacterial activity was recorded against *S. epidermidis*. The MIC of extract for *S. epidermidis* was 2 mg/ml. It was found to be 4 mg/ml for *P. aeruginosa*, *E. coli* and *S. aureus*. ZOI for *S. epidermidis* was reported to be 13.8 ± 1.9 mm. It was reported that chloramphenicol demonstrated significantly higher antimicrobial activity (ZOI = 21.8 ± 0.4 mm) compared to the extract. No sign of toxicity was reportedly exhibited by the extract.

### Other mango-based cosmeceuticals

The therapeutic effect and cosmetic importance of *M. indica* is brought into synergy in its use in cosmetic formulations such as mango lipstick and mango cream/lotion. Jain et al. [59] formulated hand-lotion and lipstick from oil derived from *M. indica* seeds. The butter was extracted from mango seeds using n-hexane as solvent. The hand lotion and formulated lipstick were assessed for their organoleptic properties which include pH, melting point, surface anomalies, spreadability and other parameters. Jain and colleagues reported that the lipstick possesses a cosmetically acceptable look with smooth texture and compatible pH while the lotion showed no phase separation. The study concluded that the formulations were comparable to synthetic chemical products. It was reported that the formulations were devoid of side effects usually associated with synthetic preparations.

Poomanee et al. [60] developed *M. indica* kernel ethanol extract-loaded anti-acne cosmeceutical. Concentration of the extract nanoemulsions was reported to be 2.5% w/w. It was optimized via response surface technology aimed at enhancing stability and skin permeation. Antibacterial activity of extract-loaded nanoemulsions against *P. acnes* was evaluated using broth micro-dilution technique. The MIC and MBC were reported to be 3.13 mg/ml and 12.60 mg/mL, respectively. The study reported that the formulation possesses excellent stability profile and exhibited satisfactory antibacterial property. Poomanee and colleagues [60] concluded that mango kernel extract nanoemulsions possess potential as delivery systems for anti-acne cosmetic products.

### Therapeutic nutraceutical formulations

Studies have shown that various parts of *M. indica* contain nutraceutical potential reportedly attributable to its possible antimicrobial activity against some food borne pathogens.

Thambi et al. [61] formulated 25 nutritional recipes from *M. indica* peel powder. Sensory properties of the recipes were assessed using five-point hedonic scale. Three different extracts were prepared from mango peel powder using acetone, aqueous and ethanol as extracting solvent, respectively. Antimicrobial effects of the different powder extracts against *E. coli, Shigella *spp, *Enterobacter *spp*, Salmonella typhus* and *Aspergillus niger strain* were conducted using the agar-well diffusion method. The study reported that acetone extract showed highest inhibitory effect against all test bacteria (ZOI = 27 mm for *E. coli*, 15 mm for *Salmonella typhus*, 13 mm for *Shigella *spp and 16 mm for *Enterobacter *spp*.*). Ethanol and aqueous extracts were reported to exhibit higher antifungal activity against *A. niger* compared to acetone extract.

The study concluded that *M. indica* peel powder possesses nutraceutical and therapeutic potentials. According to the study, this potential was demonstrated through its inhibitory effect against test organisms. Thus, mango peel powder has potential utilization for nutritional and therapeutic purposes in food and health industries.

Rawung et al. [62] developed functional cookies from fermented *M. indica* fruit and *Anredera cordifolia* (binahong) leaves. Mango is reported to contain high level of nutrients, fibre, minerals macronutrients and micronutrients. This is in addition to possession of bioactive compounds like vitamin C, beta-carotene and phytochemicals. These secondary metabolites are reported to be the basis of its wound healing property [12]. Rawung and colleagues [60] found that the cookies contain high concentration of vitamin C and significant antioxidant activity implying possession of potential wound healing activity. The researchers reported that the formulation has mean ascorbic acid content of 129.76 ± 8.13 mg/100 g and DPPH antioxidant activity of 35.15 ± 4.34. The study concluded that fermented mango and binahong possess great potential as healthy snack cookies for postoperative patients to accelerate wound healing process.

Pérez-Chabela and Hernández-Alcántara [63] investigated mango flour and flours from other edible plant parts. They reported that these flours are valuable economic alternatives for the improvement of nutritional value and functional quality of processed foods. In addition, mango and other flours assessed were found to be good sources of prebiotic fibres. The study concluded that these flours possess potential prebiotic effect, modulatory activity and effect on composition of gastro-intestinal tract micro-biota (Table 2).

**Table 2:** Summary of selected antimicrobial formulations from mango plant parts

Formulation	Mango Plant Part	Microbe of Interest	Reference
Topical gel	Leaf	*P. aeruginosa and B. subtilis*	[51]
Antimicrobial ointment	Kernel and Leaf Leaf Leaf	*S. aureus, E. coli* and *Salmonella sp* *C.albicans* *P. acnes*	[4] [49] [50]
Toothpaste	Mango peel	*S. aureus, B. cereus, P. aeruginosa* and* E. coli*	[57]
Mouthwash	Leaf Mango twig Leaf	*S. mutans, S. mitis* and *S. salivarius* *S. mutans* *E. faecalis*, *S. aureus*, *S. mutans*, *E. coli*, and *C. albicans*	[55] [54] [56]
Body scrub Liquid soap	Seed Seed	*P. aeruginosa, S. aureus* and *C. auris*	[52] [53]
Therapeutic nutraceuticals	Mango peel Mango fruit Mango fruit	*E. coli, Salmonella typhi, Shigella* spp, *Enterobacter* spp and *A. niger* Gastro-intestinal system GIT micro-biota	[61] [62] [63]
Wound healing Cosmeceuticals Anti-acne, Lipstick	Mango peel Kernel Seed	*P. aeruginosa, E. coli and S. aureus* *P. acnes*	[58] [60] [59]

### Excipients

Excipients are ingredients used in pharmaceutical product preparations. They serve as binder, disintegrant or are used for pH adjustment. There are synthetic, natural or semi-synthetic excipients. The use of plant derived excipients is fast gaining ground and opening up new avenue to solve current drug delivery issues in the pharmaceutical industry. According to Ologunagba et al. [64], natural products possess comparative advantages over synthetic polymers, hence their increasing use as excipients in formulation systems. Natural excipients include natural polymers such as pectin, gums and mucilage. Some sources of plant derived natural polymers for pharmaceutical preparations are mango peel, banana peel, mango gum, dehydrated banana powder, guar gum, gum karaya, starch, ispaghula husk, chitosan, *Lepidium sativum* mucilage and fenugreek seed mucilage.

### Pectin, gums and other plant-derived excipients

Pectins are hydrophilic polysaccharide carbohydrates. They aid in the liberation of active pharmaceutical ingredient (API) in the upper part of the digestive system through enzymatic catabolic reactions when used as an excipient [65]. Natural disintegrants are reported to have a number of advantages over synthetic disintegrants. These advantages include low cost, nontoxicity and biodegradability. Starches, mango pectin, mango gum, guar gum, soy polysaccharide, plantago seed mucilage, locust bean gum, agar and treated agar are examples of natural disintegrants [66–68].

Binders are used in pharmaceutical preparation to strengthen inter-particle bond capacity within a formulation and enable cohesion. Pectin is used in pharmaceutical formulations as binding and gelling agent [69]. It is used as a stabilizer in liquid medications and emulsions, and it increases the thickness of some pharmaceutical preparations [70]. The usefulness of pectin for various applications is dependent on its physicochemical characteristics. These include methoxyl content, the degree of esterification and galacturonic acid content. Other characteristics are sugar content, jellying characteristic, total soluble solids, moisture content, pH and colour. Assessment of arsenic concentration, microbial load and lead content is conducted to ascertain the safety of pectin for use [71]. Gums are hydrocolloids containing deliquescent molecules with strong affinity for water to form thick solutions or gels [72]. Polymeric nanocarriers are substances with small dimension and high surface area with ability to enhance permeability and dissolution of enclosed molecules. This ability enhances their use for medical applications such as imaging, diagnostic techniques and other therapeutic applications. According to Spizziri et al. [73], location-specific release of active pharmaceutical ingredient (API) with antimicrobial property spurred interest in the advancement of novel polymeric nanocarriers. Methods of extraction of natural polymers for utilization in pharmaceutical formulations include conventional acidified water-based method, microwave-assisted method, enzyme-assisted method, subcritical-assisted method and ultrasound-assisted method.

### *M. indica* utilization as an excipient

Mango peel pectin has reportedly found use as a good source of high-quality pectin extraction [68, 74]. Chaiwarat et al. [75] conducted a research aimed at formulation of clindamycin hydrochloride loaded de-esterified low-methoxyl *M. indica* peel pectin film. *M. indica* peel pectin was obtained via microwave-assisted extraction technique and de-esterified. Titration technique was used to ascertain the degree of esterification. Sodium hydroxide was used as the base in the reaction. The pectin film was prepared using remodelled ionotropic gelation with a solution-casting method. Antibacterial activities of de-esterified pectin (DP), de-esterified pectin containing clindamycin HCl (DPC) and commercial low methoxyl pectin(cLMP) films were evaluated against stock cultures of *S. aureus* and *P. acnes* using disc diffusion method. One gram (1 g) of commercial clindamycin HCl in 100 ml of pectin solution (1% w/v film forming solution) was the positive control. Film without clindamycin HCl was used as negative control. Data were expressed as mean ± standard deviation, and significance of result was analysed.

The study reported that the positive control exhibited insignificantly higher antibacterial activity against all test organisms (42.02 ± 0.52 mm for *S. aureus* and 77.18 ± 1.50 mm for *P. acnes*) in comparison with the commercial low methoxyl pectin (cLMP) and de-esterified pectin containing clindamycin HCl (DPC) films, respectively. Drug release profile of cLMP and DP was assessed. The study reported that there was no significant difference in the drug delivery profiles between commercial low methoxyl pectin and de-esterified pectin films. Further, the study stated that cLMPC and DPC films demonstrated considerable amount of drug loading content with no significant difference between the two.

Chaiwarat and colleagues [75] concluded that the de-esterified pectin obtained from *M. indica* peel has potential as a film forming agent for topical antimicrobial medications comparable to commercial low-methoxyl pectin. *M. indica* peel pectin thus has potential as an alternative to commercial low-methoxyl pectin utilized in preparation of clindamycin topical formulation commonly used for the treatment of skin infections.

Siddiqui and colleagues [65] extracted pectin from *M. indica* peel and assessed its potential as a binder in ibuprofen tablet formulation. The micrometric and post compression characteristics were examined to ascertain the size and binding property of the pectin. Mango pectin was extracted via the conventional hot water extraction technique. 50 g, 75 g, 100 g and 125 g of pectin were used for different ibuprofen tablet formulations, respectively. The researchers reported dissolution of 100 g/tablet formulation in less than 5 min. In addition, the micrometric property of the formulation was reported to demonstrate good binding and flowing abilities. The study concluded that pectin obtained from *M. indica* can be used as disintegrating agent in pharmaceutical compositions.

Ahmed and Abass [76] conducted a research to investigate *M. indica* gum (extracted from the trunk) as a sustain release polymer in glibenclamide tablets matrix. The gum was extracted using microwave heat extraction method. Binding, sustained release, disintegrating properties of the formulation were evaluated. The tablets were formulated by wet granulation technique. According to Ahmed and colleague [76], assessment of physicochemical, organoleptic and other characteristics such as swelling index, Hausner’s ratio of the formulated tablets yielded results which inferred that *M. indica* gum possesses binding property. The study concluded that tablets formulated from this gum exhibited favourable sustained drug delivery property.

Ologunagba et al. [64] conducted a study to extract and characterize mango seed endosperm gum. The extraction was carried out using the microwave-assisted technique. Phytochemical, pharmacognostic, microbial and proximate analyses were conducted. The researchers reported the following results: Swelling Index (%) 2.27 ± 0.19, Water binding capacity (%) 112.00 ± 0.10, Bulk Density (g/cm^3^) 0.51 ± 0.01, Tapped Density (g/cm^3^) 0.63 ± 0.01, Area of Repose (Aº) 29.35° ± 0.02, Compressibility Index (%) 19.10 ± 0.03 and Hausner’s ratio 1.23 ± 0.05. The study reported that the results obtained were favourable and significant. The researchers concluded that mango seed gum possesses promising binding and disintegrant properties and great potential as excipient in pharmaceutical formulations.

Gragasin et al. [71] processed and assessed *M. indica* peels as source of pectin for utilization in pharmaceutical formulations. Extraction was carried out via conventional hot water method. The study reported that percentage yield of pectin was dependent on mango peel to solvent ratio, temperature, pH and duration of extraction. The researchers noted that dried mango peel yielded higher pectin quantity. Physicochemical analysis was conducted. The pectin obtained was graded according to standard specifications. The reported result stated that mango pectin is odourless, has methoxyl content range between 12.65 and 12.84% and galacturonic acid content range between 92.82 and 98.65%. In addition, level of esterification was reported to range between 76 and 79%. The study reported that the mango pectin obtained conformed to standard USP specifications.

Malviya and Kulkanir [70] extracted and characterised pectin from mango peels. The pectin was obtained via acidified water-based extraction technique using Soxhlet apparatus. Physicochemical, phytochemical, organoleptic (colour, door, taste, fracture and texture) analyses and micrometric properties of the pectin were evaluated. Micrometric analysis included parameters such as particle size, bulk density, tapped density, true density, flow property, bulkiness, swelling index, surface tension and thickness of the mango pectin. Malviya and colleague reported that mango peel pectin exhibited good flow property, satisfactory surface tension and solubility in warm water. Binding quality of the excipient in tablets was reported to be dependent on surface tension and other parameters [70]. The study concluded that pectin obtained from mango peel has potential utilization as excipient in the preparation of oral medications such as capsules and tablets (Table 3).

**Table 3:** Summary of excipients obtained from mango plant parts

Mango plant part	Natural excipient	Use	Extraction technique	References
Peel	Pectin	De-esterified pectin film	Microwave assisted	[75]
Peel	Pectin	Binding agent in ibuprofen tablet formulation	Conventional hot water extraction	[65]
Bark/Trunk	Gum	Sustain release polymer in glibenclamide matrix tablet preparation	Microwave heat extraction	[76]
Seed	Gum	Excipient	Microwave assisted	[64]
Peel	Pectin		Conventional hot water extraction	[71]
Peel	Pectin	Binding agent	Acidified water base	[70]

### *M. indica* gum exudate

Ali and colleagues [77] reported that phytochemicals found in plants possess antiviral characteristics which can be harnessed in therapeutic management of viral diseases. The researchers reported that these phytochemicals which comprise quercetin, ellagic acid, ursolic acid, caffeic acid, thymol and caffeic acid can target viral protein and potentially inhibit viral replication. *M. indica* contains quercetin and ellagic acid in addition to other phytochemicals. Malabadi and colleagues [78] reported that mango gum together with gum exudates from other plants such as okra gum, acacia gum, tamarind gum and cashew gum was used as immunity booster and herbal remedy for throat infections, cough and common cold during second wave of COVID-19 pandemic in India. The study reported that the gums were utilized in the preparation of dietary food recipes and consumed as additional nutritional enhancement for Covid-19 patients during therapy. According to Malabadi and colleagues, consumption of the gum therapeutic diet along with regular medication aided quick restoration of health to patients during COVID-19 pandemic in India. This is a possible indication that *M. indica* gum has the potential for use in pharmaceutical preparations relating to management of viral infections.

Plant gums have found use in pharmaceutical industry for thickening and stabilizing formulations as binding, suspending and emulsifying agents and for sustained delivery of drugs [72, 79–81]. This is because natural gums have been reportedly found to possess advantages such as bio-degradability, lesser toxicity, lower cost of exploitation, abundance, flexibility and biocompatibility over synthetic excipients by researchers.

According to Kaddam et al. [82], although plant gums and mucilage have been reported to possess possible use in therapeutic formulations due to the favourable properties they possess, ingestion of high concentration is potentially toxic. Kaddam and colleagues noted that there are no significant reports of clinical evidence to support inhibition of SARS-CoV-2 viral replication load in patients by natural gums and mucilage (Tables 4, 5).

**Table 4:** Ethnomedicinal use, validation, antimicrobial activity and main phytochemical constituent of selected *M. indica* parts

Antimicrobial activity	Use in ethnic medicine	Experimental validation	Possible phytochemical	Related formulation
Antibacterial	Mango kernel, seed and leaves are used in treatment of wounds and boils in Asia and Africa	Bshabshe et al., [23] investigated the antibacterial activity of ethanol extract of mango kernel on clinical isolates of *S. aureus* and MRSA	Tannins [30, 31] Mangiferin [33, 34]	Antimicrobial ointment[4, 50] Topical gel [51]
Antifungal	Mango leaves decoction are taken orally and applied on affected areas	Disegha and Akanni [28] conducted a study on the antifungal property of *M. indica* leaf extract on *Aspergillus fumigatus, A. flavus, A. niger* and *C. albicans*	Mangiferin	Antifungal ointment [49]
Antiviral	Mango leaf, bark, fruits, flowers	Ho et al. reported antiviral activity of flavonoids against *Herpes simplex* virus, respiratory syncytial virus, *Parainfluenza* virus, and *Adenovirus* [41]	Flavonoids [83] Mangiferin	
Antimalarial	Leaves	Adepiti et al. [27] investigated the in vivo anti-malarial activity of a leaf decoction on *Plasmodium berghei* infested mice model	Flavonoids Tannins [86] Mangiferin Alkaloids	The study reported 55% *P.berghei* reduction by the fifth day of treatment
Anti-halitosis	Mango twig is used as chewing stick	Dandekar and Winnier [54] assessed activity of mango twigs extracts against *S. mutans*	Flavonoids Tannins Mangiferin	Toothpaste [57], Mouthwash [54], Mouthwash [55]
Gastro intestinal system ailment	It is used in folk medicine as an astringent, remedy for bowel obstruction, and vomiting	Pacheco-Ordaz [38] assessed the intestinal activity of mango extract	Tannins	Therapeutic nutraceuticals [61–63]

**Table 5:** Antimicrobial activity, functional group and basic nucleus/structure of some selected phytochemicals in mango

Phytochemical	Chemical name	Basic chemical nucleus/structure	Functional group	Main antimicrobial effect
Mangiferin (present in almost all parts of mango)	Xanthone glucoside	2-C-β-D-gluco-pyranosyl-1,3,6,7-tetrahydroxyxanthone	Highly reactive –OH group	Antiviral Antifungal Antimalarial Antibacterial
Tannins (present mainly in leaves, bark and seed kernel)	Gallotanins Ellagitannins, etc.		phenolic hydroxyl groups	Antibacterial
Flavonoid (present mainly in flower, fruit and leaf)	Benzo-ϓ-pyrone derivatives		hydroxyl groups Methoxy groups double bond	Antiviral
Alkaloids (in leaf, stem bark, kernel and root)	Quinines Quinolines, etc.		Basic nitrogen atom Amine groups	Antimalarial Antibacterial

## Discussion

Medicinal plant parts and extracts have been the focus of several researches. This is because there is increasing need to develop novel therapeutic and pharmaceutical products as alternatives to synthetic products. The interest in pharmacological use of medicinal plants for therapeutic purposes is intended to solve the issue of environmental contamination associated with synthetic products in addition to the growing antimicrobial resistance crisis which is a public health issue of concern. Almost every part of mango plant is used in traditional medicine for the treatment of different health issues.

This review is concerned mainly with scientific researches focused on validation of *M. indica* as an antimicrobial agent and its antimicrobial use in traditional medicine. The review also aimed at utilization of mango plant as a pharmaceutical excipient.

It has been established that phytochemicals are the scientific basis for the pharmacological activities of mango plant parts. Although it is well known that many secondary metabolites possess pharmacological abilities, the major phytochemicals attributed with these properties are the phenolic compounds. Polyphenols, a group of phenolic compounds, are abundantly present in plants. They are characterized by the presence of aromatic hydroxyl groups and are categorized according to the nature of their carbon skeleton. Several researches have been conducted to investigate pharmacological potentials of polyphenols. These potentials include antioxidant, anticancer, anti-inflammatory, anti-diabetic, antimicrobial, anthelmintic, gastroprotective, hepatoprotective, immunomodulatory and antiplasmodial properties.

Tannins, flavonoids and mangiferin are the major polyphenols reportedly known for antimicrobial activities [84]. Antimicrobial activities of phytochemicals have been found to be dependent on seasons and environmental conditions, type of solvent used in the extraction process, method of extraction, part of plant used and yield of extraction. The yield of extraction process is also dependent on the part of plant used, season, type of solvent used and the method of extraction.

Mangiferin is reported to be the most abundant phytochemical in mango plant and is found in almost all parts of the plant [34]. From the studies reviewed, mangiferin appears to exert a wider spectrum of antibacterial activity compared to other major phytochemicals due to the ability to inhibit both Gram positive and Gram-negative bacteria. Mangiferin is also shown to exert antifungal, antiviral, and antiplasmodial effects.

Tannins are concentrated in stem bark and seed kernel of *M. indica* though they can be detected in other plant parts. Generally, tannins have been found to exert relatively higher antibacterial activity when compared to exhibition of other antimicrobial properties such as antifungal effect [30]. However, the antibacterial capacity exhibited against Gram negative bacteria is limited due to their relatively large molecular size and charge. Tannins possess astringent taste; hence, they are considered defensive molecules synthesized to protect plant tissues from herbivorous attacks [85].

Flavonoids are mostly concentrated in the fruits and leaves of *M. indica*. They occur primarily as glycosides and polymers [42, 83]. Flavonoids are benzo-ϓ-pyrone derivatives consisting of phenolic and pyran rings. They are classified according to the substitutions on the rings. These substitutions include hydroxyl, methoxy and glycosidic side groups.

It is noteworthy that the effectiveness of antimicrobial activities of these phytochemicals is affected by the following: (i) Choice of solvent utilized in the extraction procedure of extracts. (ii) Extraction method. (iii) Type of test organism involved and (iv) Seasonal variations.

This was validated by findings from researches conducted by Maharaj et al. [22], Adepiti et al. [27] and Pacheco-Ordaz et al. [38]. Extraction process involving high temperature can denature some phytochemicals making them less active. According to Pacheco-Ordaz et al., alkaline solvents increase yield of phytochemicals in an extract. This is attributed to their ability to break ester bonds linking them to the cell wall components of the plant thereby releasing majority of bound secondary metabolites.

In addition, methanol extracts of mango kernel, leaf, peel, and stem bark appear to possess higher antibacterial activity against *S. aureus* compared to other solvent extracts [4, 18, 22].

Antimicrobial activity of plant part extracts is also dependent on the type of microorganism involved. Extracts containing higher concentration of tannin in comparison with other phytochemicals will be limited in antimicrobial activity against Gram-negative *E. coli* [30]. This is because tannin is unable to inhibit *E.coli* due to (1) the bacteria’s negatively charged outer membrane (2) tannin’s possession of relative high negative charge and large molecular size in contrast to other phytochemicals.

The mechanisms of action of phytochemicals described in this study corroborate and are in sync with the degree of effectiveness of selected antimicrobial formulations considered in the review.

Isolated pure phytochemicals appear to be less active compared to those in crude extracts. This was validated by Masibo and He [36] in their study. Crude extracts from *M. indica* leaf and bark demonstrated more effective pharmacological activity compared to pure isolated bioactive compounds. This is suggestive of the fact that antimicrobial activity and other pharmacological effects of these secondary metabolites involve synergy of actions. Hence, synergism of the various mango phytochemicals is important for maximum pharmacological activities. This synergism is another validation for the effective use of mango plant parts crude extracts as antimicrobial (pharmacological) agents in traditional medicine.

In addition, mango pectin has found more possible use as a pharmaceutical excipient compared to other mango plant natural polymers [65, 71, 75]. Main source of *M. indica* pectin is the fruit peel. Mango excipients are extracted via a number of methods which include microwave-assisted technique and conventional hot water extraction method. They are used in pharmaceutical formulations as binders, disintegrant, sustain release polymer and film forming agent.

## Conclusion

This study illustrated various possible mechanism of action of selected phytochemicals present in mango plant part extracts and adduced evidence from scientific studies for their utilization in antimicrobial formulations. The study also validated the use of selected mango plant parts and extracts in ethnomedicine.

This review demonstrated that understanding the characteristics and structure of bioactive compounds in plant extracts as well as their mode of action is pivotal to the utilization of the extracts in antimicrobial formulations. It can be deduced from the study that effective antimicrobial activity of mango plant extracts and formulations requires synergy of actions among various phytochemical constituents present.

Furthermore, the effectiveness of excipients derived from *M. indica* in pharmaceutical preparations is possibly related to the functional groups within the bioactive compounds in the plant extract. Further research into this is therefore necessary. It was observed that there is a dearth of knowledge concerning the mechanisms of action of phytochemicals. Hence, more researches that will be focused on discovery of new phytochemicals and their modes of action should be pursued. It is recommended that more studies aimed at unveiling new areas of utilization of *M. indica* in the pharmaceutical industry should be further explored.

## Figures and Tables

**Fig. 6:**
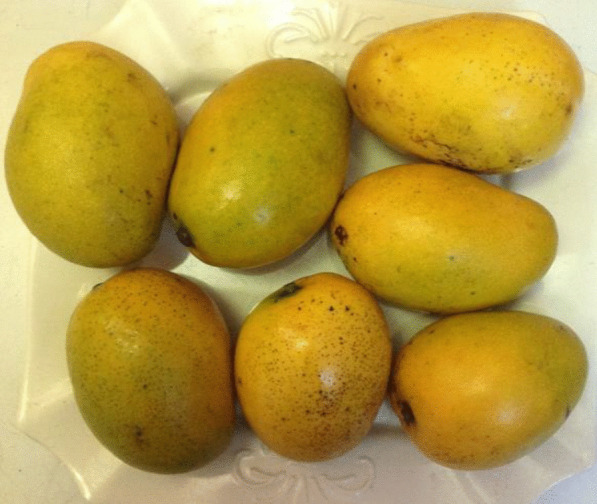
*Mangifera indica* fruits (Ogbomosho variety, Nigeria). Source: Mojisola Alaiya

**Fig. 7:**
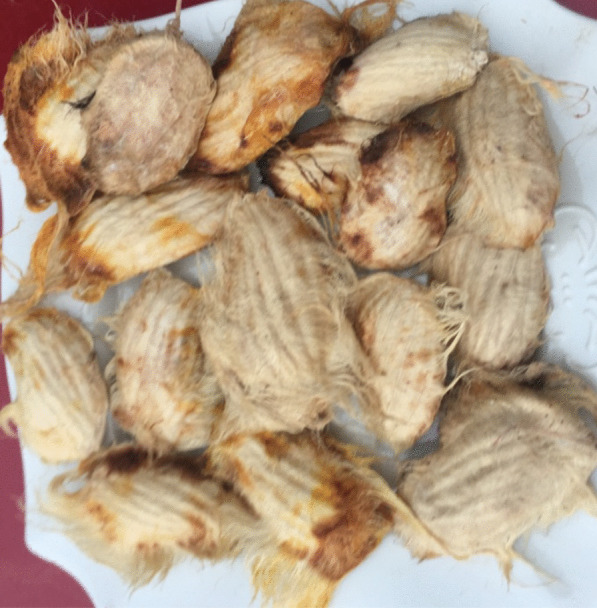
*Mangifera indica* seed kernels. Size: 85 mm by 83.8 mm. Source: Mojisola Alaiya

**Fig. 8:**
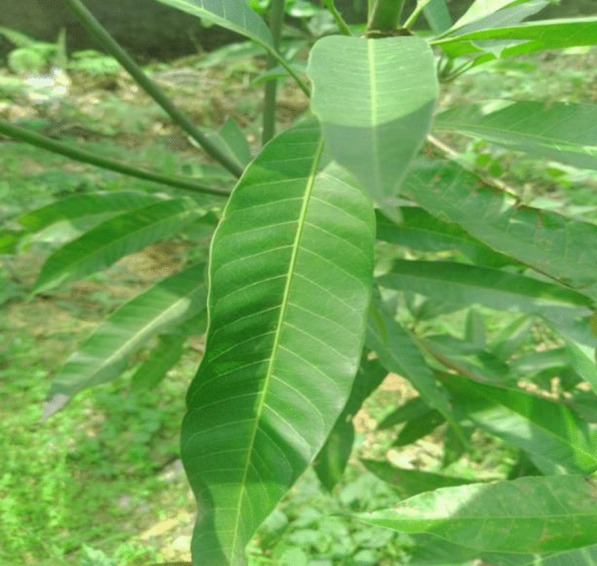
Mangifera indica leaves. Size: 79.7 mm by 83.2 mm. Source: Mojisola Alaiya

## Data Availability

Data sharing is not applicable to this article as no datasets were generated or analysed during the study.

## Appendix

See Figs. 6, 7, 8.

Several varieties of *M. indica* are cultivated in different geographical areas of the world, and these include Tommy Atkins, Ataulfo and Kent cultivars. The Ogbomosho variety is predominantly cultivated in Nigeria. The mango fruits in Fig. 1 above were harvested from a private farm in Arepo, Ogun, Nigeria during mango season. The fruits were identified and authenticated at the Herbarium, Department of Botany, University of Ibadan, Nigeria, and given voucher number UIH-22822. Mango is one of the important tropical fruit crop cultivated in the world. Mango fruits are consumed directly or processed to make other food products globally, and the seed kernels are discarded as agricultural waste. Utilising *Mangifera indica* kernels in the production of personal care antimicrobial preparations is beneficial to the health industry and as a waste management strategy.

Size: 109.2 mm by 126.2 mm.

To obtain mango seed kernels, the fruits were washed with distilled water and air dried at room temperature. The mango fruits were peeled and the flesh separated from seed using a blunt knife. The seeds were thereafter sun-dried until they attain a constant weight.

The *Mangifera indica* leaves’ image was taken at a private farm in Arepo, Ogun, Nigeria. The mango leaf was taken to the Herbarium, Department of Botany, University of Ibadan, Nigeria, for identification and authentication and was given voucher number UIH-22822.

## Abbreviations

ANOVA: Analysis of Variance
cLMP: Commercial low-methoxyl pectin
COVID: Coronavirus disease
DPC: De-esterified pectin containing clindamycin HCl
DPPH: Diphenylpicryl hydrazine
FAO: Food and Agriculture Organization
HCl: Hydrochloric acid
HPLC: High performance liquid chromatography
MBC: Minimum bactericidal concentration
MFC: Minimum fungicidal concentration
MIC: Minimum inhibitory concentration
MRSA: Methicillin-resistant *Staphylococcus aureus*
PHIC: Public health issue of international concern
QS: Quorum sensing
SARS-CoV-2: Severe acute respiratory syndrome coronavirus 2
WHO: World Health Organization
USP: United States Pharmacopeia
ZOI: Zone of inhibition
